# Unseen pain behind a smile; oral health and psychological distress among Indonesian transgenders (*warias*): a survey study on oral discomfort, sense of defeat, and oral hygiene behaviors

**DOI:** 10.3389/froh.2026.1736745

**Published:** 2026-04-15

**Authors:** Yvonne A. B. Buunk-Werkhoven, Ciptasari Prabawanti, Abraham P. Buunk, Arie Dijkstra

**Affiliations:** 1 Faculty of Medicine, Kauno University of Applied Sciences, Kaunas, Lithuania; 2 Magister of Science of Psychology, Faculty of Psychology, Universitas Ahmad Dahlan, Yogyakarta, Indonesia; 3 Faculty of Behavioural and Social Sciences, University of Groningen, Groningen, Netherlands

**Keywords:** Indonesian transgenders (*warias*), oral discomfort, oral hygiene behavior, psychological factors, sense of defeat

## Abstract

**Background:**

This study explored oral hygiene behavior (OHB), physical and psychological oral discomfort (OD), and their relationship with sense of defeat (SoD) among Indonesian transgender individuals (*warias*), for whom oral health plays a vital role in social and sexual interactions. Given the orofacial area's impact on attractiveness and intimacy, poor oral health may significantly affect the wellbeing and perceived desirability of *warias*.

**Methods:**

A cross-sectional study was conducted in March–April 2019 among 92 *warias*, with a mean age of 36 years (standard deviation = 10.5) in Jakarta, recruited via cluster sampling from five districts. Data were collected using a digital, semistructured questionnaire, including an OHB index and the Indonesian version of the Oral Discomfort Scale, which measures both psychological and physical discomfort. Sense of defeat was also assessed. The study was classified as a one-time, non-invasive procedure and was exempt from medical research regulations.

**Results:**

Correlation analyses showed that smoking was significantly positively associated with SoD (*r* = 0.25, *p* = 0.019), but not with OD or OHB. Perceived oral health was significantly positively associated with experienced *emotional* aspect (*r* = 0.31, *p* = 0.003) and with consumption of sugar-containing snacks and/or soft drinks (*r* = 0.24, *p* = 0.02), but it was significantly negatively associated with SoD (*r* = −0.24, *p* = 0.02), OD (*r* = −0.26, *p* = 0.01), and ODPhy (*r* = −0.27, *p* = 0.01). Linear regression analysis identified both SoD (*β* = 0.23, *p* = 0.023) and experienced emotional aspect (*β* = −0.30, *p* = 0.004) as significant independent predictors of OD, explaining 18% of the variance [*R*^2^ = 0.18, *F*(3,91) = 7.58, *p* = 0.001]. The variable *expectations* of optimal OHB did not have a significant effect (*β* = −0.11, ns).

**Conclusions:**

Psychological distress—particularly feelings of defeat—plays a significant role in the oral health of Indonesian *warias*. Healthcare and social service providers consider this connection and both emotional wellbeing and oral self-care in interventions.

## Introduction

Despite continued attention to the promotion of oral health, oral diseases still appear to be the most common health problems worldwide. Poor oral hygiene can negatively impact daily activities, physical health, mental wellbeing, and social health across all age groups, particularly in low- and middle-income countries and among marginalized communities (1). One understudied vulnerable population is the Indonesian *warias*, who through their sex-work activities, are at high risk of oral health problems and HIV/AIDS-related diseases (2).

The term *waria* refers to male-to-female transgender individuals in Indonesia. *Warias* frequently practice anal–oral sex in commercial and non-commercial settings, placing them at higher risk for HIV and other sexually transmitted infections (3). While kissing and non-genital licking pose no risk of infection, unprotected anal–oral sexual contact is considered a high-risk activity (4).

*Warias* can be defined as people who appear androgynous or behave androgynously, or as biological males who cross-dress and adopt the behavioral and societal roles of females. The first definition of *waria* refers to androgynous gender behavior and the second definition refers to a socially constructed identity (5). According to Nemoto, the term “transgender” simply means “people whose gender identity and gender expression differ from their biological sex” (6). Oetomo explained that many *warias* in Indonesia engage in commercial sex work (5).

Prabawanti et al. (3) found that 52% of clients of transgender sex workers (TSWs) identified as heterosexual, 41.3% as bisexual, and 6.7% as gay men. Most clients of TSWs were married (94.8%), but only 10.8% were living with their wives or regular female partners.

Findings of previous studies suggested that tailored health interventions could increase *warias*’ control over their general and HIV-related health-seeking behavior (7). From an oral health perspective, people with HIV may experience higher prevalence and severity of periodontitis, particularly as they age (8). Therefore, maintaining optimal oral hygiene is especially important to prevent health complications.

In addition, poor oral hygiene, such as unsound teeth or bad breath, can negatively impact an individual's perceived attractiveness, which is crucial for *warias*, whose social and sexual interactions—particularly in sex work—rely on physical and emotional connections or short-term relationships (9). In judgments of physical attractiveness, the orofacial area is the most important feature after the eyes (10, 11). Therefore, the importance of maintaining optimal oral hygiene and experiencing wellbeing without subjective oral discomfort should not be underestimated, particularly in the context of social interactions, including dating and the formation of intimate relationships.

Moreover, oral discomfort can increase psychological distress, affecting self-esteem and self-care practices, and further influencing the emotional and social wellbeing of *warias*. These effects may be compounded by the experience of being a *waria* in the larger social structure: *Warias* face stigma, discrimination, and marginalization. One psychological concept that may capture this is sense of defeat—the experience of being at the lower end in the societal struggle for resources (12). It is the emotional experience of loss, powerlessness, and inner surrender to adversity that can undermine the motivation for self-care and negatively impact health-seeking behaviors. Indeed, sense of defeat and social status have theoretically meaningful relations with oral self-care and oral health (13). The relationship of sense of defeat with oral discomfort is probably complex: Higher oral discomfort may contribute to higher sense of defeat, but higher sense of defeat may also contribute to oral discomfort through its negative influence on self self-care and health-seeking.

At present, there is a lack of data on oral health status and self-care, especially in relation to psychological distress in *warias*. Therefore, addressing oral health in *warias* is essential, not only for improving their oral outcomes but also for enhancing their overall quality of life, social functioning, and sexual health outcomes. In addition, oral health professionals, primary care physicians, general practitioners, and social workers should not only provide safe sex education but also integrate oral health education into preventive healthcare interventions specifically tailored to *warias*. By raising awareness and promoting oral care in tailored health interventions among *warias,* both health and oral care professionals can significantly improve the general health, oral comfort, and quality of life of *warias* (4, 8).

### Overview of present research

The overall aim of this study was to gather information to inspire and build interventions to help *warias* improve or maintain their oral health and related wellbeing (quality of life). First, we gathered descriptive data to reveal their current oral health status and problems, thereby identifying targets for interventions. Second, we statistically tested how the different aspects of oral health, oral discomfort (OD), and feelings of defeat (SoD) are related to each other. This may further guide interventions toward relevant variables. These insights can contribute to the development of a targeted oral hygiene intervention for this population. More specifically, we also assessed which variables best predicted OD, which was considered a variable of major importance.

## Materials and methods

### Study design, size, and setting

This cross-sectional human field study recruited respondents using a two-stage sampling procedure. In the first stage, five districts of Jakarta (Central, East, West, South, and North Jakarta) were selected using a cluster sampling approach to ensure geographic representation across the city. In the second stage, respondents within each selected district were recruited through convenience sampling, based on their availability and willingness to participate. The Srikandi Sejati Foundation—a community-based organization that supports *warias* in Jakarta—coordinated the recruitment of respondents by mobilizing district coordinators (*mami*) in each of the five districts.

For this cross-sectional study, permission was obtained from Siklus Indonesia, Jakarta, Indonesia, and approval was granted by the Research Ethics Committee of Universitas Katolik Indonesia Atma Jaya, Jakarta (Nr. 0521/III/LPPM-PM.10.05/04/2019). Data were collected using digital semi-structured questionnaires, in accordance with universal ethical principles and the Declaration of Helsinki. Participation was voluntarily; the *warias* were informed about what the participation entailed and that they were free to refuse to contribute. An extensive formal written informed consent was waived and only verbal informed consent was obtained. The Dutch Central Committee on Research Involving Human Subjects determined that research requiring completion of a one-time questionnaire does not fall under the scope of the Medical Research Involving Human Subjects Act (CCMO) (14).

### Participants

Based on previous studies by the second author (3, 7), eligible participants met the following inclusion criteria: (1) self-identified as *warias* (male-to-female transgender individuals); (2) aged 18 years or older; and (3) residents of one of the five districts of Jakarta included in the study. The estimated number of *warias* in Jakarta in 2019 ranged from approximately 8,000 to 12,000 individuals. For this study, a total of 92 *warias* participated in interviews conducted between late March and the first week of April 2019. Participants were recruited from five districts of Jakarta, based on recommendations from *mami* (community leaders among *warias*) and the founder of the Srikandi Sejati Foundation, a local organization that supports *waria* communities. The distribution of respondents across the districts was as follows: Central 18 (19.6%), North 14 (15.2%), and West, East, and South 20 each (21.7%). During data collection, all potential participants were invited to designated venues within their respective districts, where face-to-face interviews were conducted. Interviews were carried out individually by trained interviewers as participants arrived.

### Sample size

A power analysis was conducted to determine the sample size for this hard-to-reach target group. The computation was based on planned statistical analyses, with linear regression demanding the highest number of cases. Using conventional levels of power (0.80) and significance (0.05), and aiming to detect medium-sized effects with three predictors, at the least 77 participants were needed (G-power version 3.1.9.6).

### Questionnaire

The culturally adapted questionnaire was checked and directly translated from English into the national language Bahasa Indonesia by a native Indonesian speaker of Indonesian descent, and was also reviewed and back-translated by experts. The first page included an introductory section describing the purpose of the study and how the results would be used, followed by a few demographic questions on district, gender, age, education. The questionnaire used a combination of questions and scales, which were open-ended, multiple choice, or to be answered on bipolar adjective rating or Likert scales.

### Bias

Recruitment for this study was conducted through *mami* and one local organization, which may have led to selection and gatekeeper bias due to the overrepresentation of *warias* who were socially connected or affiliated with these communities. The relatively small sample size of 92 participants—representing only a fraction of Jakarta's estimated *waria* population—raises concerns about sampling bias and limits representativeness across the five districts. The use of self-administered and anonymous questionnaires may have introduced social desirability bias, as participants may have tailored their responses to perceived expectations rather than their actual behavior or opinions. Data collection over a short, specific period further increases the risk of temporal bias if contextual events influenced participation or responses*.*

### Measures

Questions assessed *warias*’ attitudes and behaviors related to oral healthcare, including their oral hygiene practices and frequency of visits to oral health professionals. Items also addressed tobacco use—e.g., the smoking of cigarettes or rolling tobacco—and frequency of smoking behavior.

*Warias*’ perceived oral health (OH) was assessed with the statement, “I would evaluate my own teeth with the number…,” using a verbal Ladder Scale ranging from 0 (“worst possible teeth”) to 10 (“best possible teeth”) (10), adapted from the Self-Anchoring Striving Scale (15). Two items assessed if they felt *physically* fit and healthy if their mouth was healthy too and if they felt *emotionally* better (about themselves) if their mouth was also healthy. *Warias* were asked to fill out what kind of toothbrush they used: a manual toothbrush, a powered toothbrush, or a combination of both types of toothbrushes. There was also one item concerning consumption of sugar-containing snacks and/or soft drinks.

### Oral discomfort

OD was measured using a two-dimensional scale to monitor *psychological* discomfort and *physical* discomfort (16). During the translation process of this scale from English into Bahasa Indonesia, some of the items partly drew from an Indonesian translation of the corresponding OHIP-14 items (17). An Indonesian translator checked and evaluated the final version of this Oral Discomfort Scale. Psychological discomfort (PsyOD; 6 items, Cronbach's *α* = 0.70) encompasses the affective aspects of oral discomfort, including tension, dissatisfaction, and embarrassment related to oral health condition or treatments. The following is an example: “Have you felt that life in general was less satisfying because of problems with your teeth, mouth or dentures?” The physical discomfort subscale (PhyOD; 5 items, Cronbach's *α* = 0.61) evaluates sensory experiences, such as pain and eating problems directly associated with teeth, mouth, or dentures. The following is an example: “Have you had painful aching in your mouth?” Responses were scored on a five-point Likert scale, i.e., “never” (0), “sometimes” (1), “regularly” (2), “often” (3), and “very often” (4). Moreover, 0 was defined as the maximal positive result, indicative of total absence of problems and 4 corresponded to a maximal discomfort. For each subscale, a total score was calculated for each respondent: for the PsyOD scale as the sum of six items, with a total score between zero and 24, and for the PhyOD scale as the sum of five items, with a total score between 0 and 20. The total score for each respondent was calculated as the sum of all 11 items in this OD scale (Cronbach's *α* = .79), and varied from 0 to 44. Higher sum scores indicated more frequent or intense feelings of oral discomfort, reflecting more psychological or physical discomfort.

### Oral hygiene behavior index

The index for *oral hygiene behavior* (OHB index; 18) was used to assess and evaluate OHB. As this original OHB index is designed to indicate actual or reported OHB, it is not considered a scale, and calculating reliability (Cronbach's *α*) to check the internal consistency of the items is not very meaningful and therefore unnecessary (19). The original OHB index—which is currently being further developed as a component of the MondiX-i® measure (20, 21)—consists of eight items addressing brushing teeth, interdental cleaning, and tongue cleaning. For example, the item “I brush my teeth as follows”: was supported by pictures illustrating different brushing methods. After the item scores were assigned weights, the item values were calculated and a sum score was computed. The individual OHB score served an indicator of self-reported oral hygiene self-care practices. OHB scores on this index ranged from 0 to 16. A high score indicated a high level of self-care OHB.

### Optimal oral self-care in OHB

Optimal oral self-care in the OHB was described as brushing your teeth twice a day (once after breakfast and once before going to sleep) using a soft-bristled toothbrush (or a powered toothbrush) and fluoride-containing toothpaste. Recommended practices included brushing softly without pressure. When using a manual toothbrush, brushing stepwise by making small strokes near the gum was considered most appropriate. When using a powered toothbrush, step by step all the sides; brushing for at least the automatic prescribed advice 2 min along the inside and the outside, and on the jackdaw and backward areas would be the best. In addition to brushing teeth, daily interdental cleaning, (i.e., the use of floss, toothpicks, or interdental brushes at least once a day) and tongue cleaning were also recommended and therefore included in the questionnaire (18, 22, 23). An item about the use of mouthwash was also included because this aid appears to be often used in Indonesia without professional advice. Performance of *optimal oral self-care in OHB* was assessed using three items: *actual* self-care regarding the specified optimal OHB over the past 7 days, the *intention* to perform these behaviors in the future, and *expectations* about continuing this oral self-care behavior moving forward. Responses were rated on a scale from 1 (“not at all”) to 5 (“absolutely have”).

### Sense of defeat

This characteristic was measured using the validated Sense of Defeat scale (24) that consists of 16 statements, which describe how people feel about themselves. The respondents were asked to circle the number that best described how they had felt in the last 7 days. The following are example items: “I have the feeling that others dońt respect me enough” and “I feel that I am basically a winner” (reverse code). Items were assessed on a 5-point scale (i.e., 0 = never to 4 = always). The total score of all 16 items in this Sense of Defeat scale (Cronbach's *α* = 0.88) varied from 0 to 64. Higher scores indicated stronger feelings of defeat, lower self-worth, and more frequent or intense experiences or feelings of being disrespected, hopeless, or inadequate. Elevated scores indicated that individuals felt more socially marginalized, rejected, or unsuccessful in their daily life.

### Statistical analysis

The collected data were downloaded into Excel and imported into IBM Statistical Package for Social Sciences 29.0 (SPSS Inc., Chicago, IL, USA) for further data analysis. The calculations included descriptive statistics; the data were subjected to frequency distributions, and means and standard deviations (SDs). The internal consistency of the used scales was assessed by Cronbach's alpha (*α*). Chi-square tests, *t*-tests, and one-way analyses of variance were performed to determine whether there were any significant differences in mean scores of the variables. A linear hierarchical regression analysis examined multivariate associations with oral discomfort as the dependent variable. Experienced *emotional* aspect, SoD, and expectations of optimal OHB, as predictors, were entered simultaneously into the model. Perceived OH was excluded as a predictor because it was conceptually considered a consequence. Differences were considered statistically significant at *p* < 0.05.

## Results

### Population

A total of 92 male-to-female transgender participants completed the digital questionnaire. The mean age was 36 years (SD = 10.5; range 19–67). While 17 participants (18.5%) identified as male, 75 participants (81.5%) identified as “*waria*.” In this sample, the respondents were living in five districts in Jakarta; 20 (21.7%) from the West, 20 (21.7%) from the East, 20 (21.7%) from the South, 14 (15.2%) from the North, and 18 (19.6%) from Central Jakarta. The level of education varied: 49 *warias* had completed high school (53.3%), two held a diploma or master's degree (2.2%), 15 participants had completed primary education (16.3%), and 26 had completed secondary school (28.3%).

### (Oral)#health

Among the 92 *warias*, more than a half, or 50 (54.3%), occasionally smoked, but not every week. One out of five, or 21 (22.8%), had never smoked a cigarette or rolling tobacco, not even a puff, while 13 (14.1%) had tried smoking but not adopted the habit. Eight (8.7%) reported smoking for a while, but not within the last 3 months. With regard to dietary habits, approximately two-third, or 59 (64%), of the participants reported consuming sugar-containing snacks and/or soft drinks between meals occasionally, but not daily. Thirteen (14.1%) respondents reported never consuming snacks and/or soft drinks, and an equal percentage reported doing so only once or twice a day.

The findings indicated that 54 (58.7%) *warias* had never visited a dentist and 56 (60.9%) had not visited a dental nurse in the 5 years prior to the survey. Approximately a quarter, or 22 (23.9%), reported had one or two dental visits, while 7 (7.6%) had attended three or four appointments in the last 5 years. Nine (9.8%) participants reported visiting a dentist more than five times in the past 5 years. Over one-quarter, or 24 (26.1%), of the participants reported having received orthodontic treatment (e.g., braces), while two-third, or 61 (66.3%), expressed concern about crooked and/or irregular tooth alignment. Only 10 (10.9%) *warias* reported ever bleaching their teeth.

More than a quarter, or 25 (27.2%), of the participants rated their perceived *oral* health (OH) as “the best possible teeth,” with a score of 10. Approximately a third, or 28 (30.4%), rated their teeth as “inadequate,” with a value of 5 or less, and 12 (13%) rated their own teeth as “the worst possible teeth,” with a value of zero.

Mean scores, standard deviations, and range values of the main variables—perceived OH, *experienced physical* and *emotional* aspect, OD, PsyOD, PhyOD, OHB, optimal self-care OH, and SoD—for the whole sample are presented in Table 1.

**Table 1:** Range, means, and standard deviation (SD) for the variables (*n* = 92).

Variables	Range	Mean (SD)
Perceived OH	0–10	6.56 (3.23)
Experienced *physical* fit	0–5	4.25 (1.26)
Experienced *emotional* comfort	0–5	4.05 (1.49)
Oral Discomfort	0–35	8.33 (6.91)
Psychological OD	0–16	3.74 (3.54)
Physical OD	0–11	3.10 (2.42)
Oral hygiene behavior	6–16	11.40 (2.28)
*Actual* optimal OHB	0–5	3.29 (1.46)
*Intention* to perform optimal OHB	0–5	3.70 (1.40)
*Expectations* optimal OHB	0–5	3.54 (1.44)
Sense of Defeat	0–48	17.97 (11.77)

Perceived *oral* health (OH) was generally rated as “sufficient.” Two-third, or 62 (67.4%), of the *warias* completely agreed that they only felt *physically* fit and healthy if their mouth was healthy too. Similarly, 60 (65.2%) participants *emotionally* felt a lot better (about themselves) if their mouth was healthy. In contrast, 6 (6.5%) of the *warias* completely disagreed with the statement regarding the *experienced physical* (body–mouth) aspect and 12 (13%) completely disagreed with the *experienced emotional* (psychological comfort) aspect.

### Oral discomfort

Table 2 presents the Indonesian *Oral Discomfort* scale (11 items in Bahasa Indonesia). *Warias* reported a relatively positive evaluation of their perceived oral discomfort, indicating that they experienced their mouth and teeth as satisfactory with minimal discomfort. Their mean scores on *psychological discomfort* suggested little oral discomfort related to psychological aspects. For instance, one out of five, or 18 (21%), reported to being (very) often self-conscious because of their teeth, mouth, or dentures. More than a half of the respondents, or 50 (54.3%), reported never feeling embarrassed, while one-third, or 33 (35.9%), reported sometimes feeling embarrassed because of problems with their teeth, mouth, or dentures.

**Table 2:** The Indonesian *oral discomfort* scale (Skala Ketidaknyamanan di Mulut; 11 items in Bahasa Indonesia); *psychological discomfort* (ketidaknyamanan psikologis*:* 1, 2, 3, 4, 5, 6) and *physical discomfort* (ketidaknyamanan fisik*:* 7, 8, 9, 10, 11).

Skala Ketidaknyamanan di Mulut*/ Oral Discomfort*	0	1	2	3	4
1. Pernahkah Anda menyadari keadaan diri Anda sendiri karena gigi, mulut, atau gigi palsu Anda? *Have you been self-conscious because of your teeth, mouth, or dentures?*	Tidak pernah/ *Never*	Kadang-kadang/ *Sometimes*	Secara teratur/ *Regularly*	Sering/ *Often*	Sangat sering/ *Very often*
2. Apakah Anda agak mudah tersinggung dengan orang lain karena masalah dengan gigi, mulut, atau gigi palsu Anda? *Have you been a bit irritable with other people because of problems with your teeth, mouth, or dentures?*	Tidak pernah/ *Never*	Kadang-kadang/ *Sometimes*	Secara teratur/ *Regularly*	Sering/ *Often*	Sangat sering/ *Very often*
3. Pernahkah Anda sedikit malu karena masalah dengan gigi, mulut, atau gigi palsu Anda? *Have you been a bit embarrassed because of problems with your teeth, mouth, or dentures?*	Tidak pernah/ *Never*	Kadang-kadang/ *Sometimes*	Secara teratur/ *Regularly*	Sering/ *Often*	Sangat sering/ *Very often*
4. Pernahkah Anda merasa tegang karena masalah dengan gigi, mulut, atau gigi palsu Anda? *Have you felt tense because of problems with your teeth, mouth, or dentures?*	Tidak pernah/ *Never*	Kadang-kadang/ *Sometimes*	Secara teratur/ *Regularly*	Sering/ *Often*	Sangat sering/ *Very often*
5. Pernahkah Anda merasa bahwa hidup Anda secara umum kurang memuaskan karena masalah dengan gigi, mulut, atau gigi palsu Anda? *Have you felt that life in general was less satisfying because of problems with your teeth, mouth, or dentures?*	Tidak pernah/ *Never*	Kadang-kadang/ *Sometimes*	Secara teratur/ *Regularly*	Sering/ *Often*	Sangat sering/ *Very often*
6. Pernahkah Anda merasa sulit untuk bersantai karena masalah dengan gigi, mulut, atau gigi palsu Anda? *Have you found it difficult to relax because of problems with your teeth, mouth, or dentures?*	Tidak pernah/ *Never*	Kadang-kadang/ *Sometimes*	Secara teratur/ *Regularly*	Sering/ *Often*	Sangat sering/ *Very often*
7. Pernahkah Anda merasakan luka yang menyakitkan di mulut? *Have you had painful aching in your mouth?*	Tidak pernah/ *Never*	Kadang-kadang/ *Sometimes*	Secara teratur/ *Regularly*	Sering/ *Often*	Sangat sering/ *Very often*
8. Pernahkah Anda mengalami kesulitan melakukan pekerjaan rutin karena masalah dengan gigi, mulut, atau gigi palsu? *Have you had difficulty doing your usual jobs because of problems with your teeth, mouth, or dentures?*	Tidak pernah/ *Never*	Kadang-kadang/ *Sometimes*	Secara teratur/ *Regularly*	Sering/ *Often*	Sangat sering/ *Very often*
9. Pernahkah Anda merasa tidak nyaman makan makanan apa pun karena masalah dengan gigi, mulut, atau gigi palsu Anda? *Have you found it uncomfortable to eat any foods because of problems with your teeth, mouth, or dentures?*	Tidak pernah/ *Never*	Kadang-kadang/ *Sometimes*	Secara teratur/ *Regularly*	Sering/ *Often*	Sangat sering/ *Very often*
10. Apakah Anda harus berhenti makan karena masalah dengan gigi, mulut, atau gigi palsu Anda? *Have you had to interrupt meals because of problems with your teeth, mouth, or dentures?*	Tidak pernah/ *Never*	Kadang-kadang/ *Sometimes*	Secara teratur/ *Regularly*	Sering/ *Often*	Sangat sering/ *Very often*
11. Apakah diet Anda tidak memuaskan karena masalah dengan gigi, mulut, atau gigi palsu Anda? *Has your diet been unsatisfactory because of problems with your teeth, mouth or dentures?*	Tidak pernah/ *Never*	Kadang-kadang/ *Sometimes*	Secara teratur/ *Regularly*	Sering/ *Often*	Sangat sering/ *Very often*

Mean scores on *physical discomfort* showed relatively low levels of physical oral discomfort. A quarter of the *warias*, or 24 (26.1%), reported never experiencing painful aching in their mouth, and less than two-third, or 57 (62%), experienced it sometimes. Only 5 (5.4%) reported often experiencing difficulty doing their usual jobs because of problems with their teeth or mouth. Ten (10.9%) had found it often uncomfortable to eat any foods and 5 (5.5%) reported that they regularly to often had to interrupt meals due to problems with their teeth, mouth or dentures.

### Oral hygiene behavior

Almost all participants, or 83 (90.2%), reported using a manual toothbrush, only 2 (2.2%) reported using a powered toothbrush, and 7 (7.2%) reported a combined use of both types of toothbrushes. The percentages of the items for those who reported using a manual toothbrush are presented in Table 3. The majority reported brushing their teeth twice a day, as recommended by professionals worldwide. Only three participants, who combined the use of both types of toothbrushes, brushed their teeth once a day. More than half of the sample brushed their teeth for 2 or 3 min. In addition, almost all *warias* used fluoricontaining toothpaste, more than a third cleaned their tongue daily, and one out of 10 never did so. The daily use of interdental cleaning methods, such as flossing, toothpicks, and interdental brushes, was not common. No differences in brushing details were observed between participants who used a manual toothbrush and those who used a powered toothbrush or alternated manual and powered. About a quarter reported never using mouthwash.

**Table 3:** Index for oral hygiene behavior (OHB index) for *waria*s (*n* = 83) (percent per item).

Index for oral hygiene behavior (using a *manual* toothbrush), and the item about mouthwash		
Items	Values	Percentage
- Frequency of tooth brushing	“Twice a day”	81.9
	“Once a day”	10.8
	“Not every day”	7.2
- Moments of tooth brushing	“Morning before breakfast”	83.1
	“Morning after breakfast”	44.6
	“Noon”	41
	“After diner in the evening”	72.3
	“Before going to sleep”	78.3
- Measure of force of tooth brushing	Softly (“1, 2, 3”)	57.7
	Softly/Forcefully (“4, 5”)	20.5
	Forcefully (“6, 7”)	21.7
- Duration of tooth brushing	“Two minutes” or “Three minutes”	28.9/22.9
	“Longer than three minutes” or “One minute”	22.9/16.9
	Shorter than “One minute”	8.4
- Method of tooth brushing	“Bass method”	1.2
	“Horizontal movement” or “Combination of methods”	50.6/20.5
	“Vertical movement” or “Circular movement”	15.7/12
- Fluoride toothpaste	“Toothpaste with fluoride”	88
	“Toothpaste without fluoride”	4.8
	“I don’t know”	7.2
- Interdental cleaning	“At least once a day” floss or tooth picks or	14.4/25.3 or
	interdental brushes	33.8
	“Not every day” interdental cleaning	14.5/43.4/24.1
	“Never” interdental cleaning	31.3–71.1
- Tongue cleaning	“Every day”	34.9
	“Sometimes”	54.2
	“Never”	10.8
- Mouthwash	“At least once a day”	36.2
	“Not every day”	39.8
	“Never”	24.1

### Optimal oral self-care in OHB

Mean scores for *actual* self-care relating to the specified optimal OHB over the past 7 days, *intention* to perform these behaviors in the future, and *expectations* about continuing optimal oral self-care behavior moving forward indicated that *warias* responded positively to professional recommendations. Participants demonstrated moderate to strong expectations and intentions to take optimal care of their teeth and mouth.

### Sense of defeat

The mean total score on the Sense of Defeat scale for *warias* indicated a low to moderate level of perceived defeat. This average falls toward the lower end, suggesting that *warias* experienced relatively fewer negative feelings regarding their self-respect, self-esteem, and perceived social acceptance, though not to an extreme degree.

Correlation analyses were carried out to establish the direction and magnitude of the associations between the main variables (Table 4). Analyses revealed that there were fewer or no associations for most of the sociodemographic factors; only smoking was significantly positively associated with SoD (*r* = 0.25, *p* = 0.019), but not with OD or OHB. Perceived OH was significantly positively associated with experienced *emotional* aspect (*r* = 0.31, *p* = 0.003) and with consumption of sugar-containing snacks and/or soft drinks (*r* = 0.24, *p* = 0.02). It was significantly negatively associated with SoD (*r* = −0.24, *p* = 0.02), OD (*r* = −0.26, *p* = 0.01), and ODPhy (*r* = −0.27, *p* = 0.01). *Expectations* of optimal OHB were significantly positively associated with experienced *physical* aspect (r = 0.25, *p* = 0.016), *actual* optimal OHB (*r* = 0.27, *p* = 0.01), and *intention* to perform optimal OHB (*r* = 0.28, *p* = 0.006).

**Table 4:** Intercorrelations (Pearson's) between the relevant variables.

Variables	1	2	3	4	5	6	7
1. Experienced *emotional* aspect	–						
2. Oral Discomfort	−0.38**	–					
3. Psychological OD	−0.36**	0.91**	–				
4. Physical OD	−0.30**	0.88**	0.65**	–			
5. Oral hygiene behavior	0.12	−0.23*	−0.23*	−0.11	–		
6. *Expectations* of optimal OHB	0.25*	−0.21*	−0.20	−0.17	0.26*	–	
7. Sense of Defeat	−0.23*	0.31**	0.27**	0.29**	−0.17	−0.10	–

*p < 0.05, **p < 0.01.

Finally, a linear hierarchical regression analysis was performed to examine the multivariate relationships with OD as the outcome variable. Conceptually, the experienced *emotional* aspect can be considered a predictor, while the perceived OH is better seen as a consequence. Therefore, experienced *emotional* aspect was entered as a predictor variable in the regression analysis, even though perceived OH was significantly associated with OD, though only with ODPhy. Moreover, the variables that showed a significant correlation with OD—SoD and *expectations* optimal OHB—were included as predictors in this analysis (Table 5). All variables were entered at once.

**Table 5:** Lineair hierarchical regression analysis for oral discomfort on SoD, experienced emotional aspect and expectations of optimal OHB in the total sample (*N* = 91), controlling for s1, and hierarchical regression of PsyOD and PhyOD, respectively, on SoD, experienced emotional aspect, and expectations of optimal OHB, controlling for s1.

Variables	Oral discomfort				Psychological OD				Physical OD			
	*β*	s1	*t*	*p*	*β*	s1	*t*	*p*	*β*	s1	*t*	*p*
Sense of defeat	0.23	0.056	−2.99	0.023	0.19	0.005	−1.96	0.050	0.23	0.004	2.22	0.029
Experienced *emotional* aspect	−0.30	0.464	2.33	0.004	−0.29	0.040	−2.83	0.006	−0.23	0.034	2.18	0.032
*Expectations* of optimal OHB	−0.11	0.471	−1.12	ns	−0.11	0.011	−1.12	ns	−0.09	0.034	−0.87	ns

Regression analysis showed that SoD and experienced *emotional* aspect were significant independent predictors of OD [*R*^2^ = 0.18, *F*(3,91) = 7.58, *p* = 0.001]. In this model, the variable *expectations* of optimal OHB did not have a significant effect. This model proved to be significant and accounted for 18% of the variance in OD.

To specify what part of OD was predicted by the variables, regression analyses were performed for both physical and psychological OD separately. These regression analyses accounted for 15% of the variance in PsyOD [*R*^2^ = 0.15, *F*(3,91) = 6.42, *p* = 0.001] and 11% of the variance in PhyOD [*R*^2^ = 0.11, *F*(3,91) = 5.04, *p* = 0.003]. The same variables, SoD and experienced *emotional* aspect emerged as significant independent predictors of PsyOD and PhyOD. Again, the variable *expectations* of optimal OHB did not have a significant effect.

## Discussion

This study aimed not only to determine to what extent OHB, OD, and SoD characterize the oral health of *warias* in Indonesia but also to examine the relationships between these variables. Using the PATHS model (25), the analyses clarified associations and potential causal relationships, identifying the variables that best predict OD and thereby informing targeted interventions that address both behavioral and psychosocial determinants of oral health in this population.

Overall, descriptive findings showed that most *warias* reported occasional smoking, frequent between-meal consumption of sugary snacks or drinks, and very limited use of dental services in the preceding 5 years. Despite low dental attendance, concerns about tooth alignment were common, cosmetic treatments were rare, and self-rated oral health was polarized, with many rating it as either very good or clearly inadequate, reflecting marked variability within the group. Consistent with gender differences observed between females and males (20, 26), *waria* participants, who often adopt behavioral and social roles associated with women, reported a relatively positive evaluation of their oral health, indicating an overall satisfactory perception of their mouth and teeth, with minimal discomfort. Physical discomfort among male-to-female transgender *warias* appeared relatively low compared to the higher levels reported by females in other studies (20, 26).

In this study, although correlations were low or moderate, the predictors experienced *emotional* aspect, SoD, and *expectations* of optimal OHB were related to OD. SoD was found to be positively correlated with OD, suggesting that *warias* with a stronger SoD experienced higher levels of oral discomfort. Since such feelings are often associated with helplessness, a reduced sense of control, and a poorer ability to cope with problems, this can decrease the motivation for regular and optimal oral self-care (27). Consequently, *warias* who felt discouraged are likely to perform optimal OHB less frequently and avoid dental visits. There is a risk that, over time, these patterns may worsen oral health and increase OD. This specifically highlights the importance of psychological factors in shaping oral health experiences. Regression analyses confirmed that experienced *emotional* aspect and SoD were associated with OD. These findings suggest that individuals reporting more positive emotional experiences related to oral health tended to report less oral discomfort, whereas higher feelings of defeat were associated with greater oral discomfort. Thus, given the cross-sectional design and the relatively low correlations, these associations should be interpreted as potential risk indicators rather than predictors. In general, positive emotional states may strengthen adaptability and stimulate health-promoting behaviors, including optimal OHB. Furthermore, *warias* who reported more positive emotional experiences reported better OHB and higher expectations regarding their ability or willingness to perform oral self-care. They may perceive oral complaints as less burdensome or cope better with (oral) discomfort. However, it must be noted that both measured constructs relate to behavior and showed associations, meaning that some conceptual overlap cannot be ruled out. Moreover, they do not directly correspond with perceptions, and these findings should be interpreted as behavioral correlations rather than differences in the way oral complaints are perceived or treated. With some caution, these findings may underscore the potential protective role of emotional wellbeing in the perception and management of oral health problems (28).

It is theoretically interesting that precisely experienced *emotional* aspect and SoD predicted OD, since these variables have in common that they all can be measured as aspects of the concept of subjective well-being. The relationship between SoD and oral health behavior was not significant, suggesting that *warias*’ expectations or knowledge regarding adequate OHB did not necessarily lead to better oral health outcomes. The discrepancy between knowledge and behavior is well documented in health psychology and suggests that awareness alone is insufficient to bring about behavioral change. Psychological barriers, such as a lack of motivation, stress, or feelings of defeat, may make it more difficult for *warias* to translate their expectations into consistent and optimal OHB (13, 18, 20). This cross-sectional study design does not allow causal interpretation; therefore, rejecting the pathway that SoD leads to neglect of oral self-care should be interpreted cautiously. The findings only suggest possible relationships between psychological factors and reported oral discomfort, rather than cause-and-effect mechanisms (13, 22). In both specific models, SoD and experienced *emotional* aspect remaining significant risk indicators, indicating that psychological factors influence not only the emotional dimension of OD but also the perception of physical symptoms.

The present findings align with similar research among forensic psychiatric patients (13), suggesting that in marginalized groups in present society, a sense of defeat may be a relevant variable when trying to improve oral self-care, oral health promotion, and visits to health clinics. Interventions aimed at improving OHB can benefit from addressing feelings of powerlessness and strengthening the sense of control individuals have over their oral health. Most participants identified as *warias*—defined as biological males who cross-dress and/or adopt female social and behavioral roles (5). These participants generally reported using a manual toothbrush, reflecting their reported oral hygiene practices, although this does not necessarily indicate greater attention to oral health (20, 26). It may be that their social role, which partly depends on their appearances, buffers the potential negative effects of SoD on self-care. Oral healthcare professionals can also support *warias* and other patients who experience psychological barriers to oral hygiene, such as a lack of motivation, stress, or feelings of defeat. For example, they can help by setting small, achievable goals; using persuasive oral hygiene communications to reinforce positive oral hygiene behavior (29); and creating a supportive, non-judgmental clinical environment. Such approaches can boost self-confidence and reduce barriers to maintaining regular and optimal oral hygiene. These findings may also indicate the need for referral to qualified persons or specialists for adequate treatment (30).

This study has several potential limitations. First, despite the use of validated indices and scales, the self-reported nature of the questionnaire may have introduced social desirability bias, leading the *warias* to provide responses that may not accurately reflect their actual opinions and behaviors. Nevertheless, this is typical of all self-reported studies, and the results suggest that the respondents had no problem indicating socially undesirable behaviors, like a lack of oral self-care, and experiences like feeling defeated in one’s life. Second, in comparison with Cronbach's *α* of 0.81 on the physical discomfort subscale (20), the lower internal consistency of 0.61 on the subscale ODPhy falls just below the commonly accepted threshold for acceptable reliability. The obtained scores should therefore be interpreted with caution, as measurement error can attenuate the observed relationships and reduce statistical power. This may indicate sample-specific characteristics or the small sample size (31), and suggests the need for further refinement or validation of the scale in future research. Third, the sample may not be representative of the *waria* population as a whole. Recruitment may have excluded *warias* who were concerned about their privacy and anonymity. In any case, much care was taken to obtain a representative sample by including *warias* from different parts of Jakarta and by involving the “*mami*” of the *warias* who had an extensive network of *warias* in this capital city.

The current findings highlight the potential relevance of psychological factors, particularly feelings of defeat, in relation to oral health behavior in *warias* and other socially marginalized populations. Psychological stressors and experiences of marginalization have been linked to poorer health behavior and barriers to preventive healthcare. Individuals exposed to chronic stress, stigma, or social exclusion may struggle to maintain consistent self-care routines, including oral hygiene practices (32, 33). The observed associations between *expectations* of optimal oral care and OHB, measured by the original OHB index, were correlational and do not imply causation. The findings should be interpreted with caution, as this cross-sectional design does not allow for causal inferences. Nevertheless, the OHB index can provide a structured approach to investigating relationships between oral health behavior and psychosocial variables in population research (19). Behavioral science perspectives are increasingly being used in oral health research to understand how psychological and contextual factors influence preventive practices and promotion of OHB (34, 35). These findings can support oral healthcare professionals in designing culturally sensitive promotion strategies for marginalized groups (33, 36–39). Future research should include broader sociopsychological and cultural factors, carefully adapt the OD scale to different languages and contexts, and involve more diverse populations to improve generalizability (40, 41).

To conclude, this study contributes to the growing body of research into the relationship between psychosocial factors and oral health behavior in marginalized populations. The findings highlight the complexity of oral health practices and suggest that behavioral and psychosocial conditions may be relevant factors when studying oral health patterns among vulnerable populations, such as Indonesian *warias*. Although the cross-sectional study design limits the interpretation of causal relationships, the study offers exploratory insights that can guide future research. Further research using longitudinal or mixed methods is needed to better understand these relationships and support the development of appropriate oral health promotion strategies for vulnerable populations.

## Data Availability

The datasets presented in this article are not readily available because the data are not publicly available in accordance with the consent provided by participants regarding the use of confidential data. Requests to access the datasets should be directed to Ciptasari Prabawanti, ciptasari.prabawanti@psy.uad.ac.id.
